# Co-exposure to planned power outages and wildfire PM_2.5_ and acute healthcare utilization in California: A time-stratified case-crossover study

**DOI:** 10.1371/journal.pmed.1005270

**Published:** 2026-10-27

**Authors:** Lauren Blair Wilner, Caitlin G. Jones-Ngo, Chen Chen, Bethany Kwoka, Vivian Do, Joel D. Kaufman, Laura C. Myers, Tarik Benmahrnia, Joan A. Casey

**Affiliations:** 1 Department of Epidemiology, University of Washington School of Public Health, Seattle, Washington, United States of America; 2 Scripps Institution of Oceanography, University of California, San Diego, California, United States of America; 3 Department of Population Health Sciences, University of Utah, Salt Lake City, Utah, United States of America; 4 Huntsman Cancer Institute, University of Utah, Salt Lake City, Utah, United States of America; 5 Physicians, Scientists and Engineers for Healthy Energy, Oakland, California, United States of America; 6 Department of Environmental Health Sciences, Columbia University Mailman School of Public Health, New York, New York, United States of America; 7 Department of Environmental and Occupational Health Sciences, University of Washington School of Public Health, Seattle, Washington, United States of America; 8 Department of Medicine, University of Washington, Seattle, Washington, United States of America; 9 Division of Research, Kaiser Permanente Northern California, Pleasanton, California, United States of America; 10 Irset Institut de Recherche en Santé, Environnement et Travail, UMR-S 1085, Inserm, University of Rennes, EHESP, Rennes, France; Helmholtz Munich, GERMANY

## Abstract

**Background:**

To prevent wildfires, electric utilities may preemptively de-energize power lines (public safety power shutoffs [PSPS]). These PSPS events may unintentionally harm health, particularly for those with pre-existing conditions like chronic obstructive pulmonary disease (COPD) or who rely on electricity-dependent medical equipment. Health impacts may worsen further if a wildfire occurs despite the power shutoff. To assess the effect of PSPS events, wildfire fine particulate matter (PM_2.5_) exposure, and their co-occurrence on cause-specific healthcare utilization.

**Methods and findings:**

We identified PSPS events in California from 2013 to 2019, categorizing magnitude based on number of people without power by zip code. We leveraged zip code-level average daily wildfire PM_2.5_ estimates and respiratory, COPD-specific, cardiovascular, and psychiatric emergency department visits and unscheduled hospital admissions among adults aged 20+ from the California Department of Health Care Access and Information. Using a time-stratified case-crossover design, we estimated the impact of same-day and lagged PSPS events alone, wildfire PM_2.5_ alone, and their co-occurrence on healthcare utilization, adjusting for daily temperature.

Severe PSPS events were associated with elevated odds of same-day respiratory encounters (OR = 1.06, 95% CI: 1.02, 1.11) and lag_0–3_ effects were similar. These associations were stronger for COPD. Compared to no PSPS or wildfire exposure, joint PSPS and 10 μg/m^3^ higher wildfire PM_2.5_ was associated with higher odds of respiratory encounters (1.16 [95% CI: 1.10, 1.22]), with evidence of multiplicative interaction. Associations were null for cardiovascular and psychiatric encounters.

Limitations include exposure misclassification, because we assigned exposure at the residential zip code level, aggregating circuit-level data and not accounting for individual behavior or the potential for PSPS events to impact wildfire PM_2.5_ exposure.

**Conclusions:**

PSPS events may increase risk of respiratory events requiring acute care utilization, especially when they co-occur with wildfire PM_2.5_.

## Author summary

**Why was this study done?:**

- To reduce wildfire risk during windy, dry conditions, electric utilities preemptively shut off power.
- While intended to promote public well-being, these power shutoffs may affect health, particularly for people with respiratory conditions or dependence on electricity-powered medical equipment.
- Few epidemiological studies have examined the health effects of these events.

**What did the researchers do and find?:**

- Using more than 6.6 million respiratory-related emergency department visits and hospital admissions across California zip codes from 2013 to 2019, we found increased odds of respiratory visits in the days following severe power shutoffs.
- Severe shutoffs were associated with 1.06-times higher odds of a respiratory visit, with stronger effects among people seeking care for chronic obstructive pulmonary disease (1.29).
- Co-occurring power shutoffs and wildfire smoke further heightened acute respiratory risks.

**What do these findings mean?:**

- As climate change drives more frequent wildfires and the electric grid continues to age, utilities and policymakers must weigh the health costs of these shutoffs against their wildfire-prevention benefits, and act to reduce harm when shutoffs do occur.
- This study was conducted at the zip code-level, potentially introducing exposure misclassification and confounding by individual-level behaviors.

## Abstract 3

In this time-stratified case-crossover study, Lauren Wilner and colleagues examine how planned power outages and wildfire-related air pollution are associated with acute healthcare use in California. Their findings suggest that severe power shutoffs, particularly when occurring alongside higher levels of wildfire PM2.5, are linked to increased respiratory healthcare encounters, with stronger associations observed among people with COPD.

## Introduction

Climate change has accelerated, driving temperatures higher and increasing variability in precipitation patterns. These higher temperatures and prolonged droughts increase the risk of wildfires. Simultaneously, the number of people living in areas with high wildfire risk has grown [1,2]. Coupled with fire management practices that yield more available wildfire fuel [1], these factors have led to increased wildfire frequency, severity, and population exposure in the Western United States [1–3].

Wildfires impact human health and communities through direct damage and cascading consequences [3]. Residential proximity to wildfires and the fine particulate matter (PM_2.5_) from wildfire smoke have been associated with adverse cardiorespiratory, neurological, and mental health outcomes [4–6]. Community-level consequences of wildfires include population displacement, damage and destruction of homes and infrastructure, and, power outages, including public safety power shutoffs (PSPS) initiated by utilities to prevent wildfires during periods of high winds and dry conditions [7, 8].

Alone, power outages are associated with elevated healthcare utilization for injuries, hypothermia, hyperthermia, cardiorespiratory outcomes, and mortality likely due to stress, lack of indoor thermal control, reduced emergency and other communication, interrupted medical care, and loss of power to electricity-dependent durable medical equipment (eDME) [9–11]. When the power goes out, people cannot run air filtration systems, refrigerators, air conditioners, or other electricity-dependent devices (e.g., nebulizer machines, oxygen concentrators) [9]. This can worsen indoor air quality, threaten indoor climate control, and cause stress that may precipitate adverse health outcomes. At particular risk are those reliant on eDME, as back-up batteries generally last <12 hours [12]. Oxygen concentrators, a device commonly used by those with chronic obstructive pulmonary disease (COPD), often have a battery life of 3–4 hours [12]. A narrative review found that failure of eDME, such as ventilators or other respiratory devices, was responsible for acute respiratory and cardiovascular visits across several power outage studies [9]. Beyond individual households, larger outages disrupt shared community infrastructure – including traffic signals, water and sanitation systems, pharmacies, cooling and warming centers, and healthcare facilities – amplifying the potential for population-level health harm [9].

Failing electric infrastructure has caused several of California’s most devastating wildfires. Despite PSPS, wildfires can still occur. In fact, the co-occurrence of wildfires with power outages in California increased from 2018 to 2020 [13]. Utility infrastructure may ignite wildfires when power lines come into contact with trees or dry ground–typically as a result of high winds, such as California’s strong Santa Ana and Diablo winds [14]. Wildfires caused by electrical lines can be among the most destructive [15], especially when these electrical lines serve dense population centers.

States have begun to adopt adaptive measures like PSPS to protect public safety from wildfires caused by electric infrastructure. One such measure, implemented in California beginning in 2012 and increasingly in other states, is PSPS events [16–18]. During a PSPS event, electric utilities proactively de-energize power lines to prevent them from causing or worsening wildfires [18]. No studies have evaluated the healthcare utilization impacts of PSPS events, which can result in large populations losing power for >24 hours. While PSPS events are planned, they may impact health similarly to unplanned outages.

PSPS events may also pose an amplified threat when wildfires still occur despite the shutoff, exposing individuals to the adverse impacts of losing power and wildfire smoke exposure. This may happen if a PSPS event is (1) used to prevent a wildfire from worsening, or (2) initiated to attempt to reduce already high wildfire risk, but a wildfire still occurs nearby. The hazard of co-occurring power outages and high concentrations of PM_2.5_ may have synergistic negative health impacts that exceed the sum of their individual effects.

To our knowledge, no epidemiological studies have evaluated this co-exposure. Certain subpopulations may be more vulnerable due to social and biologic risk factors. For example, those living with COPD, who often rely on oxygen concentrators, may face disproportionate risks during power outages and exceptional risk when wildfire smoke is also present.

This study aimed to assess the association between PSPS events, wildfire PM_2.5_ exposure, and their co-occurrence and respiratory, COPD, cardiovascular, and psychiatric acute healthcare utilization in California from 2013 to 2019.

## Methods

### Study population

In this zip code-level time-stratified case-crossover [19] study in California, we used data on emergency department visits and unscheduled hospital admissions among adults ≥20 years of age from 2013 to 2019 from the California Department of Health Care Access and Information (HCAI) [20], across 1,504 California zip codes where we had access to power outage data and at least one healthcare encounter occurred (97% of zip codes) (S1 Fig). Based on findings in prior power outage and wildfire literature, we studied all-cause respiratory, COPD, cardiovascular, and psychiatric healthcare encounters [3,9]. This study was approved by the California Health and Human Services Agency’s Committee for the Protection of Human Subjects and informed consent was waived (project number 2021-116). Analyses for this study were planned via Zoom meetings with the study team. We initially explored three exposure metrics: the absolute and relative metrics presented in the manuscript, as well as a percentage of customers-out metric, which was dropped due to collinearity with the absolute and relative metrics. Initial statistical analyses included same-day PSPS exposure and 3-day average wildfire PM_2.5_ exposure, but in response to reviewer comments, we included only same-day PSPS and same-day wildfire PM_2.5_ exposure in the main analysis.

### Exposures and covariates

#### PSPS data.

We measured exposure to PSPS events from 2013 to 2022 using data from PSE Healthy Energy, which compiled historic PSPS events from three of California’s major energy providers, Pacific Gas and Electric (PG&E), San Diego Gas and Electric (SDG&E), and Southern California Edison (SCE) [16,18]. These three utility companies cover 88% of electrical customers in California [21,22].

The PSPS dataset contains circuit-level information for each PSPS event across these three major utilities. A circuit is a network of electrical lines that deliver electricity from a power source to a defined service area of customers. A PSPS event may impact multiple circuits, so each circuit-event had a start time, end time, duration, and number of customers impacted [16–18]. Utility companies may not de-energize an entire circuit, and a circuit may be energized and de-energized multiple times throughout an event. Because we lacked information on these two characteristics of outages, we assumed that the number of people affected were evenly spread across the impacted circuit and were without power for the full duration of the circuit-event. Given the small geographic area covered by a circuit, exposure misclassification is likely minimal.

We overlaid these circuit-level data containing the number of customers affected [16,18] with high-resolution gridded estimates of population from the 2020 census in California [23] to estimate the number of people theoretically served by each circuit. We then determined the number of customers impacted on a circuit and proportionally split them between zip codes. We used this information to derive our exposure of interest, the total number of customers impacted each day of a PSPS event in each zip code (referred to as our absolute metric in this paper). We examined both (1) same-day PSPS event exposure and (2) same-day + prior 3-days (lag_0–3_, rolling average for days 0–3) PSPS event exposure. We chose this lag period because while most COPD exacerbations occur quickly (same-day), some may be delayed (median of 4 days). For this reason, as well as the long duration of PSPS events, we assessed exposure over a 4-day period [24].

We classified event severity by the number of customers without power because outages affecting more people impose broader disruption including on shared community infrastructure, making them more likely to produce population-level health effects [9]. Based on tertiles of these counts for each of these, we categorized the magnitude of PSPS events at the zip code level for both same-day and lag_0–3_ exposure as mild, moderate, or severe for each zip code-event (Table 1). Most events exceeded a day, and the median duration was 86 hours. The PSPS tertiles for both exposure metrics were calculated at the zip code-event level to equally weight longer and shorter events and base the magnitude of severity on number of customers without power rather than duration. We also expected reduced exposure measurement error when moving from mild to moderate to severe PSPS events, as more people within a specified zip code were truly exposed during severe events.

**Table 1:** Exposure categories. PSPS exposure zip code-level magnitude classification cutoffs. The absolute metric represents the number of customers out. The relative metric measures the relative magnitude of severity of the PSPS event and is the product of the total number of customers without power and the proportion of customers affected. PSPS, public safety power shutoff.

Exposure window	Metric	Mild	Moderate	Severe
Same-day	Absolute (customers out)	**≤**61	62–593	>593
Same-day	Relative	**≤**1	2–145	>145
Lag_0–3_	Absolute (customers out)	**≤**45	46–458	>458
Lag_0–3_	Relative	**≤**1	2–139	>139

Because of highly variable population counts within zip codes, we also constructed a secondary metric meant to measure the relative magnitude of severity of the PSPS event (referred to as our relative metric). This metric was computed by multiplying the total number of customers without power by the proportion of customers affected in each zip code (S1 Methods). We again created tertiles of exposure at the zip code-event level (S1 Methods). Our primary analysis focuses on the absolute metric for the simplicity of interpretation.

Finally, in a secondary analysis, we constructed a metric of PSPS duration. To do this, we calculated a lag_0–6_ day PSPS exposure as the total number of hours without power by zip code in the week prior to the index or referent day.

#### Wildfire smoke data.

We used daily wildfire PM_2.5_ estimates at the zip code level [25]. The estimates were modeled using a set of machine learning models with multiple predictor variables, including air quality monitor data, aerosol optical depth, land cover, and meteorological conditions, to produce daily PM_2.5_ concentrations [25]. Wildfire-specific PM_2.5_ was then calculated by imputing non-wildfire-related PM_2.5_ concentrations using a chained random forest algorithm and taking the difference between these values and modeled PM_2.5_ concentrations. When comparing the observed PM_2.5_ concentrations across all US Environmental Protection Agency Monitoring sites versus modeled PM_2.5_ concentrations, Aguilera and colleagues reported *R*^2^ = 0.83. We used same-day wildfire PM_2.5_ exposure for same-day models, and lag_0–3_ averaged wildfire PM_2.5_ exposure for lag_0–3_ models.

#### Temperature data.

We linked gridded (4 km grid) daily maximum temperature data from gridMET extracted at the location of zip code population-weighted centroids to the index or referent day as a confounder [26].

### Outcomes

Our outcome data relied on HCAI individual-level emergency department visits and unscheduled (unplanned/emergency) hospital admissions from 2013 to 2019, which included date of admission, associated diagnoses based on *International Classification of Diseases, Ninth Edition* (*ICD*-9) and Tenth Edition (*ICD*-10) codes, hospital zip code, and patient’s residential zip code. When a patient’s zip code was missing, we used the zip code of the hospital (*n* = 10,727 [0.09%] cases).

Using primary diagnosis codes, we identified combined daily counts of emergency department visits and unscheduled hospital admissions for the following causes: all-cause respiratory, COPD, cardiovascular, and psychiatric (S1 Table). Diagnostic codes outside the primary position were not used. We chose these cause groups based on previous research of power outages and health [9]. We excluded scheduled hospitalizations (e.g., planned/elective admissions) and visits among those 19 years and younger. As such, our analysis includes emergency department visits without hospitalization and acute hospital admissions, the latter typically originating in the emergency department. We refer to emergency department visits and hospital admissions as encounters for ease of reading throughout this paper.

### Statistical analysis

This study examined the association between PSPS events alone, same-day and lag_0–3_ wildfire PM_2.5_ alone, and their joint impacts on all-cause respiratory, COPD, cardiovascular, and psychiatric emergency department encounters and hospitalizations. We assessed same-day and lag_0–3_ exposures. Patient index and referent days in this case-crossover study were linked to exposure data using zip code and date. The date of emergency department visit or hospital admission was considered the index day. We selected referent days for every index day by matching on zip code, day of week, month, and year. In other words, if an index encounter occurred on a Monday in January 2015, all other Mondays in January 2015 *except* for the day of the hospital encounter, were considered referent days.

The outcome was whether a case (ED visit or hospitalization) occurred for an individual on that day. For each day with at least one case (index day), we identified matched referent days within the same calendar month (all same weekdays) and created one matched stratum for each case. We then compared PSPS exposure on index days versus on referent days within the zip code where the individual with the case lived using conditional logistic regression while accounting for the matched strata. Each individual served as their own control across time.

Case-crossover studies are commonly used to evaluate short-term exposures and acute outcomes, as they inherently control for time-invariant confounding factors; the time-stratified referent days control for day-of-week and seasonality [19]. The primary analysis examined the effects of severe PSPS events using an absolute exposure metric and continuous wildfire PM_2.5_. Secondary analyses examined mild and moderate PSPS events, a relative PSPS exposure metric, and continuous wildfire PM_2.5_. Because PSPS events typically last more than 24 hours, we performed this analysis between both same-day and lag_0–3_ PSPS and wildfire PM_2.5_ exposure and healthcare encounters.

Using a single conditional logistic regression model for each health endpoint, we examined the association between (1) a PSPS event alone, (2) wildfire PM_2.5_ alone, and (3) co-occurring PSPS event and wildfire PM_2.5_ exposure and cause-specific emergency department visits and hospital admissions, accounting for the matched strata of index days and referent periods (more details and equation in S1 Methods). Each model included PSPS event exposure, wildfire PM_2.5_ exposure modeled using a linear term per 10 μg/m^3^, an interaction term between PSPS event and wildfire PM_2.5_ exposure to assess multiplicative interaction, and a natural cubic spline with three degrees of freedom on same-day maximum temperature (selected based on best model fit to account for the potential non-linear relationship between temperature and health outcomes) (S1 Methods). We assessed co-exposure in two ways: (1) multiplicative scale interaction effect - the deviation from the expected combined effects of PSPS and wildfire PM_2.5_ on a multiplicative scale; and (2) joint effect - total risk of both hazards when they co-occurred (S1 Methods). In secondary analyses, we ran all models for three separate age groupings: (1) all ages over 20 years, (2) 20–64 years old, and (3) 65+ years old.

To account for the possibility that longer PSPS events may have greater health impacts, in our secondary analysis, we ran a model with a linear term for total number of hours without power in the prior week (lag_0–6_) as our main PSPS effect. We accordingly parameterized wildfire PM_2.5_ as a lag_0–6_ day average for consistency. All modeling methods were otherwise the same.

R version 4.4.2 and Python version 3.13.0 were used for all analyses. Code is available at https://github.com/laurenwilner/psps_ca_analysis. Artificial Intelligence tools were not used for any part of this study or manuscript. This study is reported as per the Strengthening the Reporting of Observational Studies in Epidemiology (STROBE) guideline (S1 Checklist).

## Results

### Health data description

This study included emergency department visits and unplanned hospital admissions from 2013 to 2019 among California residents ≥20 years old (Tables 2 and S2). We studied four endpoints: respiratory encounters (*n* = 6,682,821), COPD encounters (*n* = 516,427), cardiovascular encounters (*n* = 2,913,338), and psychiatric encounters (*n* = 1,943,709). Overall, 68.7% of encounters were emergency department visits, while 31.3% were unplanned hospital admissions (Table 2).

**Table 2:** Emergency department and unplanned hospitalization counts. Emergency department visits (without hospitalization) or unplanned hospital admissions by type of encounter from California Department of Health Care Access and Information from 2013 to 2019. COPD, chronic obstructive pulmonary disease.

	All-cause respiratory *n* (%)	COPD *n* (%)	Cardiovascular *n* (%)	Psychiatric *n* (%)
Emergency department visit	5,599,535 (83.8%)	303,988 (58.9%)	1,306,387 (44.8%)	1,071,198 (55.1%)
Hospital admission	1,083,286 (16.2%)	212,439 (41.1%)	1,606,951 (55.2%)	872,511 (44.9%)

During the 7-year study period, there were 11,293 zip code-days with PSPS events (Fig 2), and most were classified as ‘severe’. Overall, 876 of the 1,721 zip codes in California experienced at least one PSPS event in the study period (S1 Fig). Most PSPS events occurred in the summer and fall (S2 Fig).

We examined our secondary exposure, wildfire PM_2.5_, independent from and concurrent with PSPS events. Among index days, the mean wildfire PM_2.5_ during a severe PSPS event was 3.30 μg/m^3^, while the mean wildfire PM_2.5_ on non-PSPS days was 0.50 μg/m^3^. Among referent days, the mean wildfire PM_2.5_ during a severe PSPS event was 3.06 μg/m^3^, while the mean wildfire PM_2.5_ on non-PSPS days was 0.50 μg/m^3^. The average wildfire PM_2.5_ concentration during index days among all four outcome categories was generally similar (S3 Table, Fig 1). There were 1,229,438 (27%) zip code-days where there was any wildfire PM_2.5_ from 2013 to 2019 and 7,656 zip code-days with any wildfire PM_2.5_ and a PSPS event (Fig 2).

**Fig 1:**
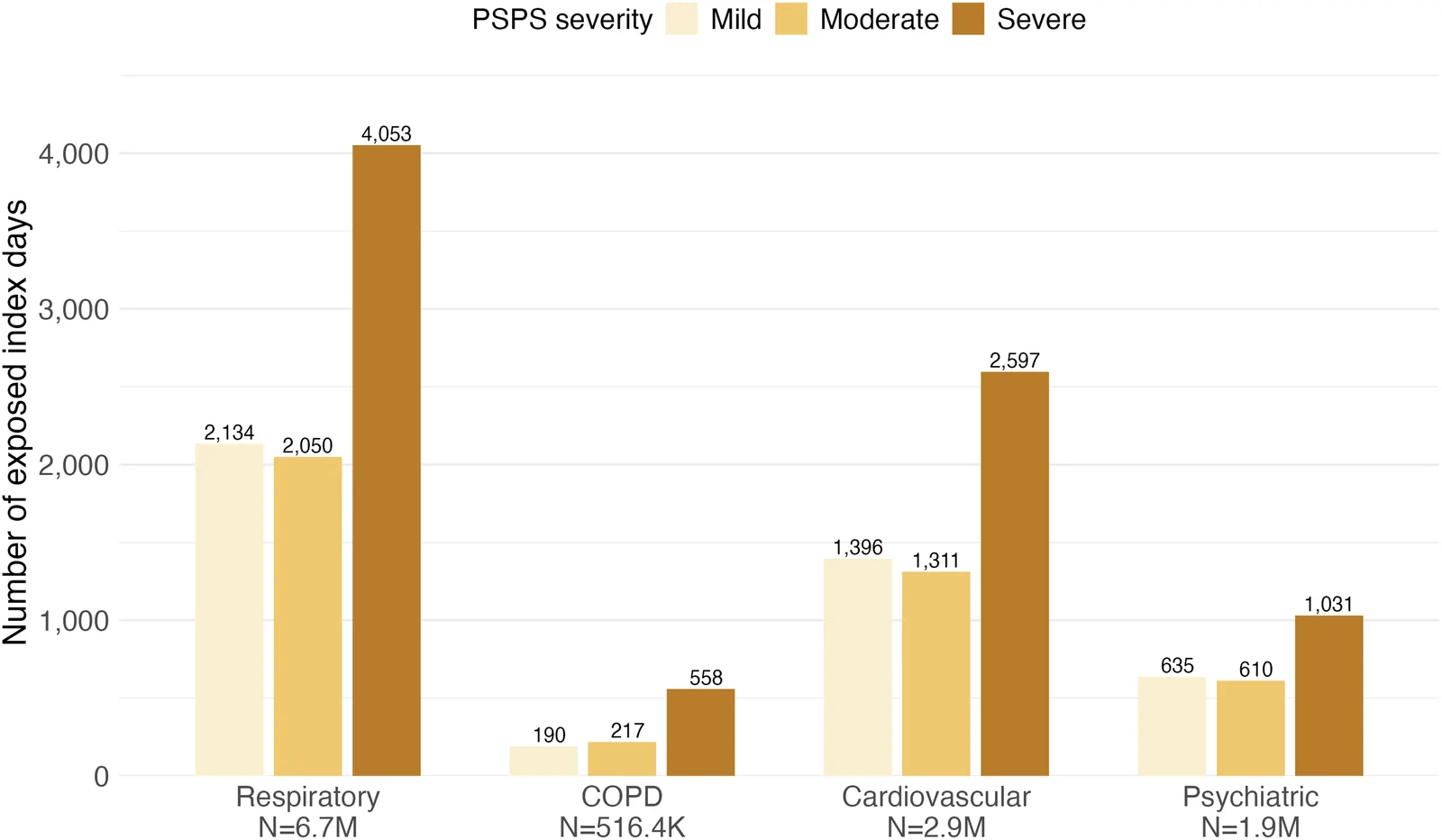
Index days by same-day exposure status. Index days were days when a healthcare encounter for all-cause respiratory, COPD, cardiovascular, or psychiatric endpoints occurred. Wildfire PM_2.5_ exposure represents the mean wildfire PM_2.5_ concentration on that day. PSPS event magnitude (mild/moderate/severe) was classified using tertiles. PSPS events were classified as mild for those affecting ≤61 people, moderate for those with >61 and ≤593 customers without power, and severe for those with >593 customers without power. COPD, chronic obstructive pulmonary disease; PSPS, public safety power shutoff.

**Fig 2:**
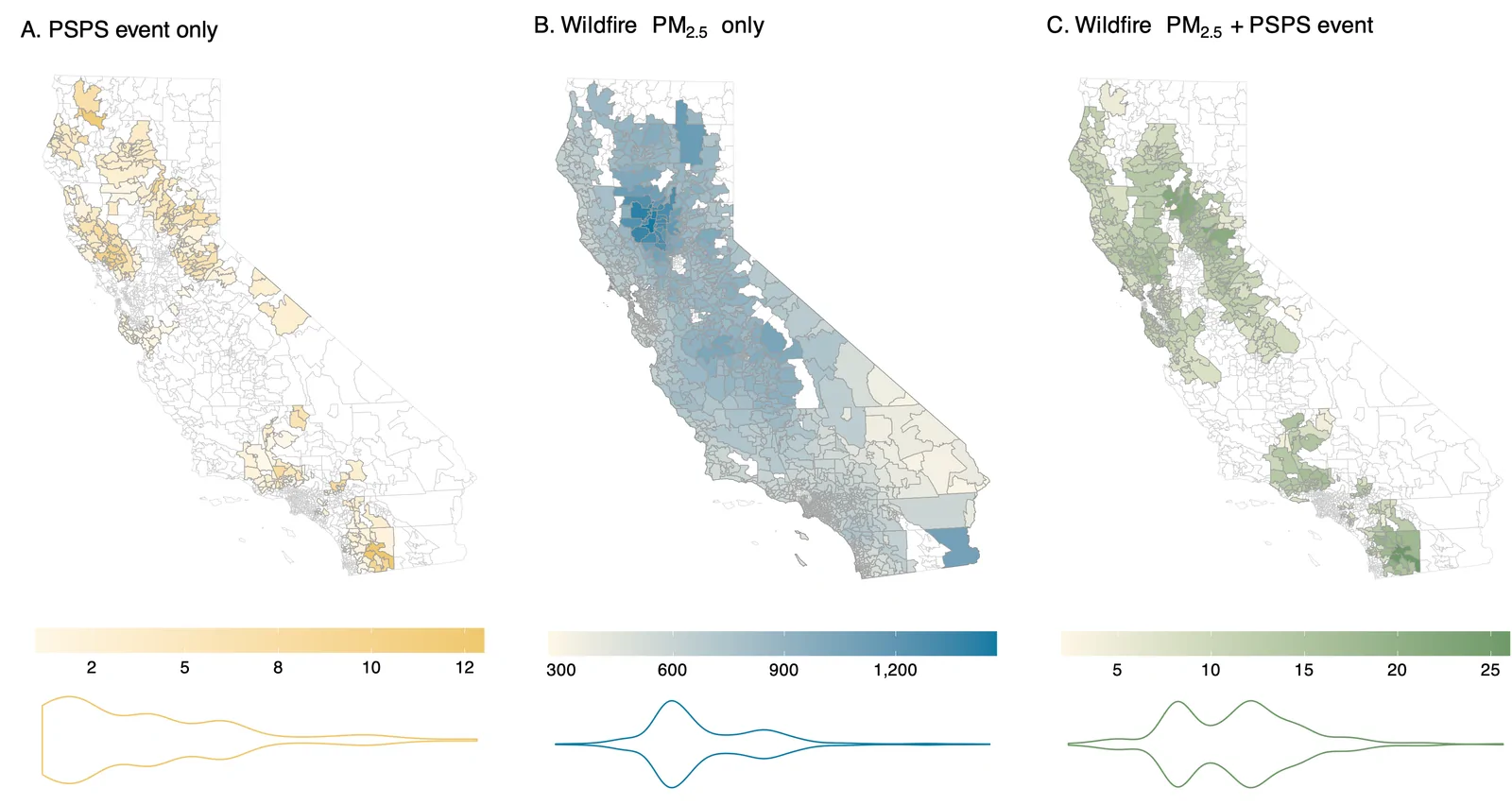
Exposure assessment for PSPS events and wildfire PM_2.5_. Number of days with **(A)** PSPS events alone, (B) wildfire PM_2.5_ alone, and (C) joint PSPS–wildfire PM_2.5_ exposure from 2013 to 2019 by California zip code. These maps are mutually exclusive, meaning that the left panel shows number of days with *only* PSPS events, the middle panel shows number of days with *only* wildfire PM_2.5_, and the right panel shows days when joint exposure occurred. Zip-days were defined as having wildfire PM_2.5_ when wildfire PM_2.5_ was >0 μg/m^3^. This study included zip codes served by the three major energy providers in California – PG&E, SDG&E, and SCE comprising approximately 88% of California electrical customers [18]. Basemaps from the U.S. Census Bureau (https://www.census.gov/geographies/mapping-files/time-series/geo/tiger-line-file.2018.html#list-tab-790442341). PSPS, public safety power shutoff.

We identified 8,237 respiratory, 965 COPD, 5,304 cardiovascular, 2,276 psychiatric index days exposed to a same-day PSPS event (mild, moderate, or severe) (S3 Table, Fig 1, lagged values in S4 Table).

### Respiratory morbidity

We found that days with severe magnitude PSPS events and no wildfire smoke were associated with an elevated same-day odds of a respiratory emergency department visit or hospital admission (absolute: OR = 1.06, 95% CI: 1.02, 1.11) (Fig 3). Respiratory emergency department visits or hospital admissions were not elevated following lag_0–3_ PSPS exposure.

**Fig 3:**
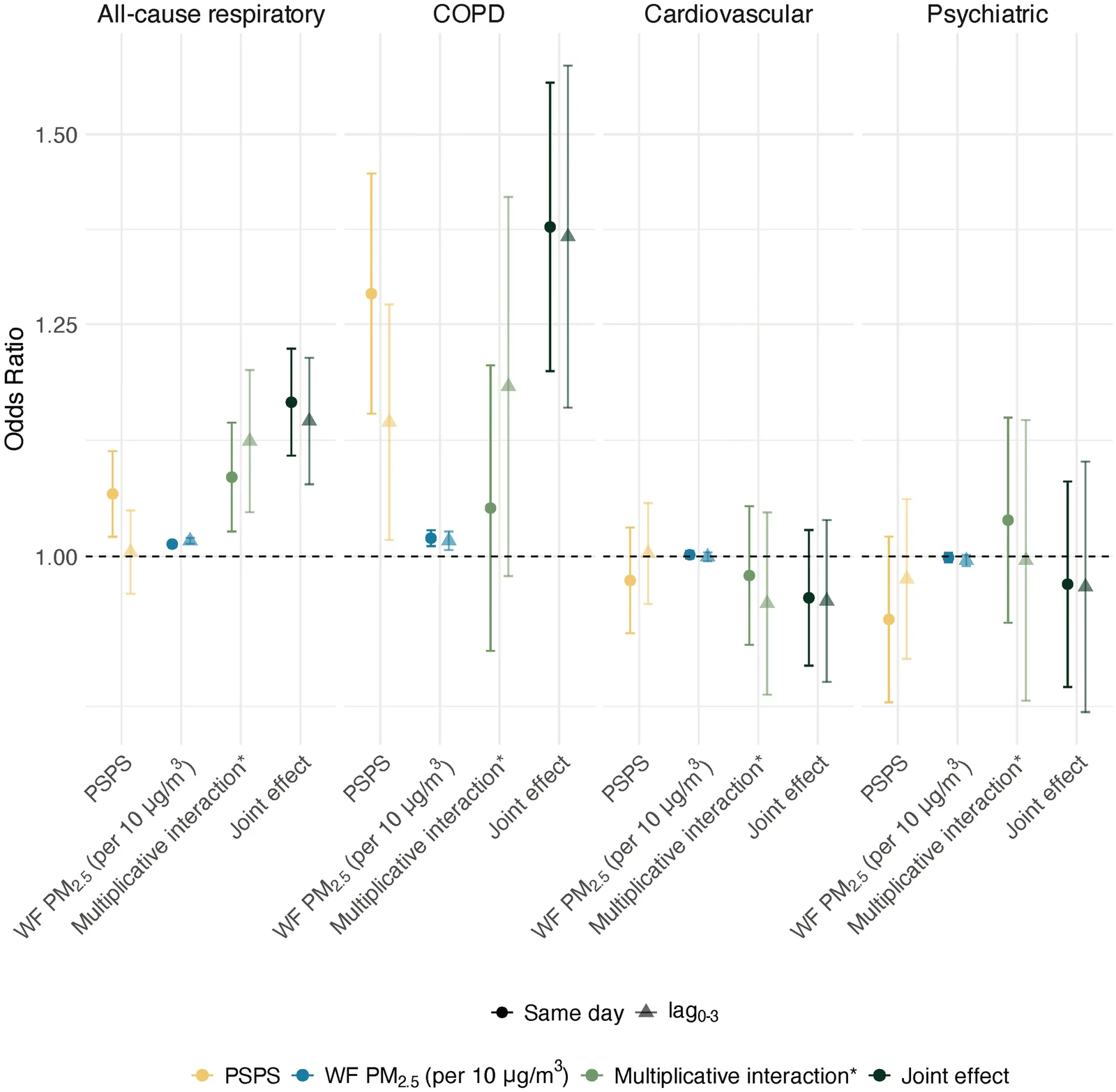
Association between severe PSPS events emergency department visits and hospital admissions. Odds ratios and 95% confidence intervals for the association between zip code-level severe PSPS events and all-cause respiratory-, COPD-, cardiovascular-, and psychiatric-related emergency department visits and hospital admissions in California from 2013 to 2019. Estimates are from separate conditional logistic regression models for each outcome. All models include PSPS event exposure, wildfire PM_2.5_ exposure, an interaction term between PSPS event and wildfire PM_2.5_ exposure, and a spline on mean daily temperature. Exposures are parameterized as same-day or lag_0–3_, with exposures averaged over lag_0–3_. COPD, chronic obstructive pulmonary disease; PSPS, public safety power shutoff. *Note: The estimate for the interaction effect represents the additional multiplicative effect of joint exposure to wildfire PM_*2.5*_ *and PSPS event on the outcome of interest. It should not be interpreted as an odds ratio in isolation, but is rather a ratio of odds ratios.*

A 10 μg/m^3^ increase in same-day and lag_0–3_ average wildfire PM_2.5_, in the absence of PSPS events, were each associated with higher odds of a respiratory emergency department visit or hospital admission (same-day: OR = 1.01 [95% CI: 1.01, 1.01], lag_0–3_: OR = 1.02 [95% CI: 1.01, 1.02]) (Fig 3).

Considering the joint effects, compared to no PSPS event and no wildfire PM_2.5_ exposure, we estimated that same-day severe PSPS event co-occurring with 10 μg/m^3^ higher wildfire PM_2.5_ was associated with higher odds of a respiratory emergency department visit or hospital admission (1.16 [95% CI: 1.10, 1.22]) (Fig 3). We found evidence of multiplicative interaction between severe PSPS events and wildfire PM_2.5_ on respiratory emergency department visits or hospital admissions (1.08 [95% CI: 1.02, 1.14]) (Fig 3). Results were similar for lag_0–3_ exposures, where we detected evidence of multiplicative interaction between severe PSPS events and wildfire PM_2.5_ on respiratory emergency department visits or hospital admissions (1.12 [95% CI: 1.04, 1.20]) (Fig 3).

When we isolated COPD encounters, we observed stronger PSPS associations. We estimated that those exposed to a same-day severe magnitude PSPS event had 1.29 (95% CI: 1.15, 1.44) times higher odds of a COPD-related emergency department visit or hospital admission (Fig 3). Lagged effects were similar, but smaller in magnitude (1.14 [95% CI: 1.02, 1.27]).

For COPD encounters alone, compared to those exposed to neither a magnitude PSPS event nor wildfire PM_2.5_, exposure to co-occurring severe PSPS event and 10 μg/m^3^ higher wildfire PM_2.5_ was associated with higher odds of emergency department visits or hospital admissions (same-day OR = 1.37, 95% CI: 1.19, 1.58; lag_0–3_ day OR = 1.36, 95% CI: 1.15, 1.60 Fig 3). There was potential evidence of multiplicative interaction between severe PSPS events and wildfire PM_2.5_ exposure that was more apparent for lag_0–3_ exposures (1.18 [95% CI: 0.98, 1.41]).

Respiratory main effects were consistent across all age strata, however, evidence of multiplicative interaction was present for those ages 20–64 years, but not in older adults (S3, S5 and S7 Figs). When isolating COPD, the effect of severe PSPS event, as well as the joint effects of severe PSPS event and wildfire PM_2.5_, were stronger in adults ages 65 years and older (S3, S5 and S7 Figs).

### Cardiovascular morbidity and psychiatric disorders

We did not observe an association between same-day or lag_0–3_ mild, moderate, or severe magnitude PSPS events alone, wildfire PM_2.5_ exposure alone, or co-occurring PSPS events and wildfire PM_2.5_ exposure and cardiovascular-related or psychiatric disorder-related emergency department visits or hospitalizations (Figs 3 and S1). This was consistent in age-stratified models as well (S3, S5 and S7 Figs).

### Secondary analysis

In a secondary analysis, we assessed a relative metric of exposure to severe PSPS events (S3 and S4 Tables), Fig 1, and results were similar to those using the absolute exposure metric (S4, S6 and S8 Figs). We also examined a relative metric for mild and moderate magnitude PSPS events, and neither they nor their interaction with wildfire PM_2.5_ exposure were associated with any type of emergency department visit or hospital admission (S4, S6 and S8 Figs).

In a secondary analysis using a lag_0–6_ day PSPS duration metric, we did not observe an association between PSPS duration and any healthcare encounters. We found modest evidence of multiplicative interaction between 8-hour longer PSPS duration and wildfire PM_2.5_ for respiratory- and COPD-related encounters for those under 65 years of age (respiratory OR = 1.01, 95% CI: [1.00, 1.01]; COPD OR = 1.01, 95% CI: [1.00, 1.03]) (S9 Fig). These results were null for older adults (S9 Fig).

### Sensitivity analysis

In a sensitivity analysis where PSPS exposure was parameterized as a continuous measure of total customers impacted, we found that for each additional 1,000 customers without power during a PSPS event, odds of all-cause respiratory emergency department visits or hospital admissions increased (same-day: OR = 1.02 [95% CI: 1.01, 1.02], lag_0–3_: OR = 1.01 [95% CI: 1.00, 1.01]) (S10 Fig). When we isolated COPD encounters, as in primary analyses, associations were stronger (same-day: OR = 1.06 [95% CI: 1.04, 1.07], lag_0–3_: OR = 1.02 [95% CI: 1.00, 1.05]) (S10 Fig). Consistent with primary analyses, associations for continuous PSPS event exposure were null for cardiovascular- and psychiatric-related emergency department visits or hospital admissions.

## Discussion

As California’s wildfire frequency and severity have increased, electric utilities have increasingly initiated more preemptive power shutoffs (PSPS events) to reduce fire risk and spread [27]. In California from 2013 to 2019, we found severe magnitude PSPS events were associated with same-day all-cause respiratory and COPD-specific, but not cardiovascular or psychiatric, emergency department visits and hospital admissions. Wildfire PM_2.5_ exposure alone was also only associated with same-day all-cause respiratory and COPD-specific encounters. The association between PSPS events and respiratory endpoints was stronger in the presence of wildfire PM_2.5_ exposure, especially for COPD.

While both power outages and wildfire smoke are associated with adverse health outcomes, their joint effects are understudied, despite a rise in co-occurrence [13] and potential for more than additive health effects. Several studies found that power outages appear to amplify the health effects of co-occurring extreme heat, storms, wind events, and floods [11,28–31]. We found evidence of multiplicative interaction between PSPS events and wildfire PM_2.5_ on respiratory healthcare utilization, particularly for COPD. The higher joint effect for COPD was driven by a stronger relationship between PSPS events and COPD encounters than all-cause respiratory encounters. The multiplicative interaction was of similar magnitude for COPD and all-cause respiratory encounters. These findings suggest that climate-driven hazards may be risk multipliers for power outage events.

Limited large-scale epidemiological studies have evaluated the relationship between any type of power outage and health [9–12,32,33]. Generally, health risks appear to rise moderately following power outages, with some evidence that the highest risk is concentrated among those using eDME [9–12,32,34]. In the days following a power outage, most, but not all [11,35], studies in New York State and Florida have found increased risk of emergency department visits or hospitalizations for respiratory illness [31,36,37], COPD [31,33], cardiovascular disease [37], food- and water-borne disease [37], injury [10], renal disease [36], and carbon monoxide poisoning [10]. Our results for severe magnitude PSPS events and respiratory encounters align with most existing literature, as do our null results for cardiovascular disease [11,35], suggesting power outages result in acute respiratory, more often than cardiovascular, disease. We found no association with psychiatric encounters, despite participants in two qualitative studies reporting increases in worry, anxiety, and stress following a power outage [38]. Although people report worry around PSPS events [39], their symptoms may not rise to a level warranting emergency psychiatric medical care.

The population health literature around PSPS events remains sparse [15,39]. Motor vehicle crashes appear largely unchanged during these events [15] but no studies have evaluated other health outcomes. PSPS events differ from other power outages in their widespread and prescribed nature. The California Public Utilities Commission requires utilities to alert customers of PSPS events in advance, potentially preventing adverse health events that may occur during unplanned outages. Because numerous states now initiate PSPS events, future work must evaluate their impacts on health to inform cost-benefit decision-making.

Prior studies have found individuals reliant on eDME, older adults, and those with versus without pre-existing conditions are at higher risk following power outage exposure [9,12,15,28,37,40]. PSPS events likely pose particular danger to COPD patients, given their reliance on eDME, sensitivity to temperature and humidity changes, and frequent comorbidities [9]. Our findings are consistent with this; we found the strongest associations between PSPS events and emergency department visits and hospitalizations were for COPD encounters, particularly among older adults. A large portion (>10%) of those with COPD rely on long-term oxygen therapy [41,42]. In New York State, power outages were associated with increased risk of COPD-related hospitalization, and power outages appear to mediate the association between severe weather and COPD healthcare utilization [33,43]. Because COPD affects an estimated 4.4% of Californians [44], future studies should focus on this population and protecting their health during PSPS and wildfire smoke events.

Short-term wildfire PM_2.5_ exposure has been associated with increased risk of respiratory hospitalizations and ED visits, but not consistently with cardiovascular encounters [5]. Studies have also identified associations between short-term wildfire PM_2.5_ exposure and COPD hospital encounters [6,45–47]. Results are mixed for mental health-related healthcare utilization, with some evidence of same-day increases [48–50]. Our study generally agrees with this existing literature.

The majority of epidemiological studies on power outages have constructed binary power outage metrics. These metrics defined a power outage as a percentage of individuals without power (e.g., 5%) within a specific geographic unit [10,11,31,33,35,37]. Others considered everyone in an affected area as exposed (e.g., New York during the 2003 blackout) [36,51]. One study used outage data at specific skilled nursing facilities [52].

Our power outage exposure assessment deviated in several key ways from previous studies. First, we evaluated only PSPS events, which are always planned, proactive cuts to power. The decision to initiate a PSPS event reflects balancing of the societal risks stemming from wildfires and the costs of the PSPS event itself [27]. Power utility companies are required to inform customers in advance and take precautions to mitigate the impacts of the outage, such as providing access to Community Resource Centers where individuals can charge devices and access WiFi [15]. Historically, PSPS events last longer than other types of power outages. Importantly, unlike previous studies, our primary exposure metric was based on counts of customers without power, rather than duration. This is because nearly all PSPS events lasted longer than 8 hours, the cutoff generally used in previous health studies [10,13]. We found that only severe PSPS events, those impacting > 593 customers, were associated with acute healthcare encounters. In a secondary analysis, PSPS duration appeared a less pertinent exposure, as we found null effects nearly universally with a duration-only based exposure, suggesting household count affected is a key health-relevant component of PSPS exposure.

This study had several limitations. The exposure assessment leveraged power utility data at the circuit level, we lacked individual-level exposure data. Despite circuit-level exposure assessment being a major improvement on power outage exposure assessment, our assumption that customers were evenly distributed across electrical circuits risks introduction of exposure misclassification. To minimize this, future studies should consider linking individual household location information if available. A second limitation of our exposure assessment is that we aggregated circuit-level data to the zip code level, leading to some exposure misclassification. Further, we assigned exposure at the residential zip code level but could not ensure that an individual was home during a PSPS event. Individuals may relocate to access power during PSPS events, potentially increasing smoke exposure when traveling outdoors. Finally, our null findings for mild and moderate events may reflect greater exposure misclassification in these categories. Because event severity is defined by the number of people experiencing outages, zip codes classified as exposed to mild or moderate events have fewer residents who actually lost power compared to zip codes exposed to severe events [53]. Importantly, we cannot determine whether exposure misclassification was correlated with wildfire smoke levels. Such correlation could arise if, for example, rural areas with larger circuits (and thus greater misclassification) also experienced higher smoke concentrations, though we lack data to assess this possibility. If present, such correlation would not necessarily bias results toward the null.

Second, the average duration of PSPS events spanned multiple days. To account for this, we examined same-day and lag_0–3_ day exposures. Future studies may wish to consider more varied lagged effects of PSPS events and wildfire PM_2.5_ exposure.

Third, our study period (2013–2019) spanned a time when PSPS events were relatively new and less frequent. Further, in recent years, multiple major California wildfires have been linked to failing utility infrastructure, including the Dixie Fire (2nd largest, caused by PG&E power lines) and the Eaton Fire (5th deadliest, suspected to be caused by SCE equipment), yet neither had PSPS events initiated. This underscores the need for further study of the interplay between wildfire events, PSPS events, and population health.

Fourth, we captured only individuals aged 20 years and older who visited an emergency department or hospital. Individuals who seek healthcare may systematically differ from those who do not. Outside hospital mortality was also not accounted for. Additionally, PSPS events may delay care-seeking behavior, and while we only assessed same-day visits, future research should consider longer periods. Because effects in children may differ, future studies should include information on their healthcare.

Fifth, our healthcare utilization categories were broad (respiratory, COPD, psychiatric, and cardiovascular). This may explain our null results for psychiatric endpoints. There is wide discrepancy in care-seeking behavior for some psychiatric endpoints, such as depression compared to illicit drug use. Further, we grouped emergency department visits with hospital admissions, however, the decision to seek care in these settings differs. Patients or families decide to seek care in the case of emergency department encounters, whereas an admitting physician decides to admit a patient. Future studies may wish to disaggregate the types and causes of encounters.

Further, we could not differentiate emergency department and inpatient admission visits due to data limitations, which may have masked different patterns in these two care settings.

Sixth, healthcare utilization may be disrupted by PSPS events. While hospitals have backup generators, they may still be at risk when diesel cannot be refueled, or generators do not supply sufficient power for all hospital operations. Utilities should coordinate more closely with California healthcare systems when deploying PSPS events [54].

Finally, in an observational study, unobserved confounding may remain. Our case-crossover design effectively handled time-invariant confounding, but short-term time-varying confounders beyond daily ambient temperature, which we controlled for, may remain.

In the US, the prevalence of wildfires and co-occurring power outages is increasing [13,55]. Since 2022, states other than California have also implemented PSPS events, including Washington, Oregon, Idaho, and Hawaii [16] in response to growing wildfire risk. Here we provide an analysis of these co-occurring hazards related to health events that require acute care utilization. As the US population ages and more individuals rely on eDME, understanding the respiratory health implications for PSPS events alone and alongside wildfire PM_2.5_ will allow mitigation strategies that reduce rather than elevate risks to human health.

## Supporting information

S1 Checklist Strengthening the Reporting of Observational Studies in Epidemiology (STROBE) guideline checklist of items that should be included in reports of case-control studies. Available at: https://doi.org/10.1097/EDE.0b013e3181577654.
(PDF)

S1 Methods Additional methodological details on on exposure assessment and statistical modeling. (PDF)

S1 Table International Classification of Disease, Ninth Edition (ICD-9) and Tenth Edition (ICD-10) codes. International Classification of Disease codes used in analysis by medical condition. COPD, chronic obstructive pulmonary disease.
(PDF)

S2 Table Demographic characteristics. Demographic characteristics of emergency department visits or hospitalizations included in the study in California from 2013 to 2019. Each observation represents a single visit, so individuals with multiple visits appear multiple times. COPD, chronic obstructive pulmonary disease.
(PDF)

S3 Table Index days by same-day exposure status. Index days are days when a healthcare encounter for all-cause respiratory, COPD, cardiovascular, or psychiatric endpoints occurred. Wildfire PM_2.5_ exposure represents the mean wildfire PM_2.5_ concentration on that day. PSPS event magnitude (mild/moderate/severe) were classified using tertiles for both absolute and relative metrics. For our absolute (primary) metric, mild events were those affecting ≤61 people, moderate were those where the number of customers without power was >61 and ≤593, and severe was defined as events where > 593 customers were without power. For our relative (secondary) metric, magnitude was classified as mild when our relative metric was ≤1, moderate when 1 < relative metric ≥145, or severe when our relative metric was >145. COPD, chronic obstructive pulmonary disease; PSPS, public safety power shutoff.
(PDF)

S4 Table Index days by lag_0–3_ day exposure status. Index days are days when a healthcare encounter for all-cause respiratory, COPD, cardiovascular, or psychiatric endpoints occurred. Wildfire PM_2.5_ exposure represents the mean wildfire PM_2.5_ concentration for lag_0–3_ days. PSPS event magnitude (mild/moderate/severe) were classified using tertiles for both absolute and relative metrics. For our absolute (primary) metric, mild events were those affecting ≤45 people, moderate were those where the number of customers without power was >45 and ≤458, and severe was defined as events where > 458 customers were without power. For our relative (secondary) metric, magnitude was classified as mild when our relative metric was ≤1, moderate when 1 < relative metric ≥ 145, or severe when our relative metric was >145. COPD, chronic obstructive pulmonary disease; PSPS, public safety power shutoff.
(PDF)

S5 Table Association between PSPS events and healthcare utilization. Odds ratios and 95% confidence intervals for the association between zip code-level severe PSPS events and all-cause respiratory-, COPD-, cardiovascular-, and psychiatric-related emergency department visits and hospital admissions in California from 2013 to 2019. Estimates are from four separate conditional logistic regression models. All models include PSPS event exposure, lag_0–3_ day wildfire PM_2.5_ exposure, an interaction term between PSPS event and wildfire PM_2.5_ exposure, and a spline on mean daily temperature. COPD, chronic obstructive pulmonary disease; PSPS, public safety power shutoff. *Note: The estimate for the interaction effect represents the additional multiplicative effect of joint exposure to wildfire PM_*2.5*_ *and PSPS event on the outcome of interest. It should not be interpreted as an odds ratio in isolation, but is rather a ratio of odds ratios.*
(PDF)

S6 Table Unadjusted association between PSPS events and healthcare utilization. Odds ratios and 95% confidence intervals for the unadjusted association between zip code-level severe PSPS events and all-cause respiratory-, COPD-, cardiovascular-, and psychiatric-related emergency department visits and hospital admissions in California from 2013 to 2019. Estimates are from four separate conditional logistic regression models. All models include PSPS event exposure, lag_0–3_ day wildfire PM_2.5_ exposure, an interaction term between PSPS event and wildfire PM_2.5_ exposure. COPD, chronic obstructive pulmonary disease; PSPS, public safety power shutoff. *Note: The estimate for the interaction effect represents the additional multiplicative effect of joint exposure to wildfire PM_*2.5*_ *and PSPS event on the outcome of interest. It should not be interpreted as an odds ratio in isolation, but is rather a ratio of odds ratios.*
(PDF)

S1 Fig Study zip codes. Spatial distribution of the 1,504 zip codes in California included in this study. There were 1,504, 1,464, 1,500, 1,501 zip codes included for all-cause respiratory-, COPD-, cardiovascular-, and psychiatric-related emergency department visits and hospital admissions. COPD, chronic obstructive pulmonary disease.
(PDF)

S2 Fig Seasonality of PSPS events and wildfire PM_2.5_ exposure. Number of zip code-event days of PSPS events and mean wildfire PM_2.5_ exposure, by month, in California during the study period (2013–2019). The number of zip code-event days of PSPS events reflects the sum over the 7-year period, while the mean wildfire PM_2.5_ exposure represents means for each month over the 7-year study period. PSPS, public safety power shutoffs.
(PDF)

S3 Fig Association between mild PSPS events and healthcare utilization by age group. Odds ratios and 95% confidence intervals for the association between zip code-level mild PSPS events (absolute metric) and all-cause respiratory-, COPD-, cardiovascular-, and psychiatric-related emergency department visits and hospital admissions in California from 2013 to 2019 by age group. Estimates are from eight separate conditional logistic regression models, including one for each health endpoint – exposure metric combination. All models include PSPS event exposure, lag_0–3_ day wildfire PM_2.5_ exposure, an interaction term between PSPS event and wildfire PM_2.5_ exposure, and a spline on mean daily temperature. The absolute metric reflects the number of individuals exposed to a PSPS event, while the relative metric reflects the severity of the event. COPD, chronic obstructive pulmonary disease. *Note: The estimate for the interaction effect represents the additional multiplicative effect of joint exposure to wildfire PM_*2.5*_ *and PSPS event on the outcome of interest. It should not be interpreted as an odds ratio in isolation, but is rather a ratio of odds ratios. PSPS, public safety power shutoff; COPD, chronic obstructive pulmonary disease.*
(PDF)

S4 Fig Association between mild PSPS events and healthcare utilization by age group (secondary exposure metric definition). Odds ratios and 95% confidence intervals for the association between zip code-level mild PSPS events (relative metric) and all-cause respiratory-, COPD-, cardiovascular-, and psychiatric-related emergency department visits and hospital admissions in California from 2013 to 2019 by age group. Estimates are from eight separate conditional logistic regression models, including one for each health endpoint – exposure metric combination. All models include PSPS event exposure, lag_0–3_ day wildfire PM_2.5_ exposure, an interaction term between PSPS event and wildfire PM_2.5_ exposure, and a spline on mean daily temperature. The absolute metric reflects the number of individuals exposed to a PSPS event, while the relative metric reflects the severity of the event. COPD, chronic obstructive pulmonary disease. *Note: The estimate for the interaction effect represents the additional multiplicative effect of joint exposure to wildfire PM_*2.5*_ *and PSPS event on the outcome of interest. It should not be interpreted as an odds ratio in isolation, but is rather a ratio of odds ratios. PSPS, public safety power shutoff; COPD, chronic obstructive pulmonary disease.*
(PDF)

S5 Fig Association between moderate PSPS events and healthcare utilization by age group. Odds ratios and 95% confidence intervals for the association between zip code-level moderate PSPS events (absolute metric) and all-cause respiratory-, COPD-, cardiovascular-, and psychiatric-related emergency department visits and hospital admissions in California from 2013 to 2019 by age group. Estimates are from eight separate conditional logistic regression models, including one for each health endpoint – exposure metric combination. All models include PSPS event exposure, lag_0–3_ day wildfire PM_2.5_ exposure, an interaction term between PSPS event and wildfire PM_2.5_ exposure, and a spline on mean daily temperature. The absolute metric reflects the number of individuals exposed to a PSPS event, while the relative metric reflects the severity of the event. COPD, chronic obstructive pulmonary disease. *Note: The estimate for the interaction effect represents the additional multiplicative effect of joint exposure to wildfire PM_*2.5*_ *and PSPS event on the outcome of interest. It should not be interpreted as an odds ratio in isolation, but is rather a ratio of odds ratios. PSPS, public safety power shutoff; COPD, chronic obstructive pulmonary disease.*
(PDF)

S6 Fig Association between moderate PSPS events and healthcare utilization by age group (secondary exposure metric definition). Odds ratios and 95% confidence intervals for the association between zip code-level moderate PSPS events (relative metric) and all-cause respiratory-, COPD-, cardiovascular-, and psychiatric-related emergency department visits and hospital admissions in California from 2013 to 2019 by age group. Estimates are from eight separate conditional logistic regression models, including one for each health endpoint – exposure metric combination. All models include PSPS event exposure, lag_0–3_ day wildfire PM_2.5_ exposure, an interaction term between PSPS event and wildfire PM_2.5_ exposure, and a spline on mean daily temperature. The absolute metric reflects the number of individuals exposed to a PSPS event, while the relative metric reflects the severity of the event. COPD, chronic obstructive pulmonary disease. *Note: The estimate for the interaction effect represents the additional multiplicative effect of joint exposure to wildfire PM_*2.5*_ *and PSPS event on the outcome of interest. It should not be interpreted as an odds ratio in isolation, but is rather a ratio of odds ratios. PSPS, public safety power shutoff; COPD, chronic obstructive pulmonary disease.*
(PDF)

S7 Fig Association between severe PSPS events and healthcare utilization by age group. Odds ratios and 95% confidence intervals for the association between zip code-level severe PSPS events (absolute metric) and all-cause respiratory-, COPD-, cardiovascular-, and psychiatric-related emergency department visits and hospital admissions in California from 2013 to 2019 by age group. Estimates are from eight separate conditional logistic regression models, including one for each health endpoint – exposure metric combination. All models include PSPS event exposure, lag_0–3_ day wildfire PM_2.5_ exposure, an interaction term between PSPS event and wildfire PM_2.5_ exposure, and a spline on mean daily temperature. The absolute metric reflects the number of individuals exposed to a PSPS event, while the relative metric reflects the severity of the event. COPD, chronic obstructive pulmonary disease. *Note: The estimate for the interaction effect represents the additional multiplicative effect of joint exposure to wildfire PM_*2.5*_ *and PSPS event on the outcome of interest. It should not be interpreted as an odds ratio in isolation, but is rather a ratio of odds ratios. PSPS, public safety power shutoff; COPD, chronic obstructive pulmonary disease.*
(PDF)

S8 Fig Association between severe PSPS events and healthcare utilization by age group (secondary exposure metric definition). Odds ratios and 95% confidence intervals for the association between zip code-level severe PSPS events (relative metric) and all-cause respiratory-, COPD-, cardiovascular-, and psychiatric-related emergency department visits and hospital admissions in California from 2013 to 2019 by age group. Estimates are from eight separate conditional logistic regression models, including one for each health endpoint – exposure metric combination. All models include PSPS event exposure, lag_0–3_ day wildfire PM_2.5_ exposure, an interaction term between PSPS event and wildfire PM_2.5_ exposure, and a spline on mean daily temperature. The absolute metric reflects the number of individuals exposed to a PSPS event, while the relative metric reflects the severity of the event. COPD, chronic obstructive pulmonary disease. *Note: The estimate for the interaction effect represents the additional multiplicative effect of joint exposure to wildfire PM_*2.5*_ *and PSPS event on the outcome of interest. It should not be interpreted as an odds ratio in isolation, but is rather a ratio of odds ratios. PSPS, public safety power shutoff; COPD, chronic obstructive pulmonary disease.*
(PDF)

S9 Fig Association between 1-week duration PSPS event exposure and healthcare utilization by age group. Odds ratios and 95% confidence intervals for the association between zip code-level PSPS event duration and all-cause respiratory-, COPD-, cardiovascular-, and psychiatric-related emergency department visits and hospital admissions in California from 2013 to 2019 by age group. Estimates are from four separate conditional logistic regression models – one for each health endpoint. All models include PSPS duration, lag_0–6_ day wildfire PM_2.5_ exposure, an interaction term between PSPS duration and wildfire PM_2.5_ exposure, and a spline on mean daily temperature. *Note: The estimate for the interaction effect represents the additional multiplicative effect of joint exposure to wildfire PM_*2.5*_ *and PSPS event on the outcome of interest. It should not be interpreted as an odds ratio in isolation, but is rather a ratio of odds ratios. PSPS, public safety power shutoff; COPD, chronic obstructive pulmonary disease.*
(PDF)

S10 Fig Association between number of customers impacted by a PSPS event and healthcare utilization by age group. Odds ratios and 95% confidence intervals for the association between zip code-level PSPS customers impacted (continuous) and all-cause respiratory-, COPD-, cardiovascular-, and psychiatric-related emergency department visits and hospital admissions in California from 2013 to 2019 by age group. Estimates are from four separate conditional logistic regression models – one for each health endpoint. All models include PSPS customers impacted, lag_0–6_ day wildfire PM_2.5_ exposure, an interaction term between PSPS duration and wildfire PM_2.5_ exposure, and a spline on mean daily temperature. *Note: The estimate for the interaction effect represents the additional multiplicative effect of joint exposure to wildfire PM_*2.5*_ *and PSPS event on the outcome of interest. It should not be interpreted as an odds ratio in isolation, but is rather a ratio of odds ratios. PSPS, public safety power shutoff; COPD, chronic obstructive pulmonary disease.*
(PDF)

## Abbreviations

COPD: chronic obstructive pulmonary disease
CPUC: California Public Utilities Commission
eDME: electricity-dependent durable medical equipment
HCAI: Health Care Access and Information
PG&E: Pacific Gas and Electric
PM2.5: Fine particulate matte
PSPS: public safety power shutoffs
SDG&E: San Diego Gas and Electric
SCE: Southern California Edison
