# Supplementary material for: Co-exposure to planned power outages and wildfire PM2.5 and acute healthcare utilization in California: A time-stratified case-crossover study
Source: PLoS Med. 2026 Oct 27;23(10):e1005270. doi: 10.1371/journal.pmed.1005270 (PMC13712598; doi:10.1371/journal.pmed.1005270)
Supplement: Supplementary file 1 [file pmed.1005270.s001.pdf]

S1 Checklist. STROBE Statement—Checklist of items that should be included in reports of ***case-control studies***. Available at: DOI: 10.1097/EDE.0b013e3181577654

	Item No	Recommendation	Section/paragraph No
**Title and abstract**	1	(*a*) Indicate the study's design with a commonly used term in the title or the abstract	Abstract, para 2
		(*b*) Provide in the abstract an informative and balanced summary of what was done and what was found	Abstract, para 2-4
**Introduction**			
Background/rationale	2	Explain the scientific background and rationale for the investigation being reported	Introduction, para 1-8
Objectives	3	State specific objectives, including any prespecified hypotheses	Introduction, para 8
**Methods**			
Study design	4	Present key elements of study design early in the paper	Materials and methods, section 0.1, 0.4
Setting	5	Describe the setting, locations, and relevant dates, including periods of recruitment, exposure, follow-up, and data collection	Materials and methods, section 0.1-0.3
Participants	6	(*a*) Give the eligibility criteria, and the sources and methods of case ascertainment and control selection. Give the rationale for the choice of cases and controls	Materials and methods, section 0.1; Results, section 0.5
		(*b*) For matched studies, give matching criteria and the number of controls per case	Materials and methods, section 0.4
Variables	7	Clearly define all outcomes, exposures, predictors, potential confounders, and effect modifiers. Give diagnostic criteria, if applicable	Materials and methods, section 0.1-0.3
Data sources/ measurement	8*	For each variable of interest, give sources of data and details of methods of assessment (measurement). Describe comparability of assessment methods if there is more than one group	Materials and methods, section 0.2-0.3
Bias	9	Describe any efforts to address potential sources of bias	Materials and methods, section 0.4
Study size	10	Explain how the study size was arrived at	Materials and methods, section 0.3
Quantitative variables	11	Explain how quantitative variables were handled in the analyses. If applicable, describe which groupings were chosen and why	Materials and methods, section 0.2-0.3
Statistical methods	12	(*a*) Describe all statistical methods, including those used to control for confounding	Materials and methods, section 0.4
		(*b*) Describe any methods used to examine subgroups and interactions	Materials and methods, section 0.4
		(*c*) Explain how missing data were addressed	Materials and methods, section 0.4
		(*d*) If applicable, explain how matching of cases and controls was addressed	Materials and methods, section 0.4
		(*e*) Describe any sensitivity analyses	Materials and

			methods, section 0.4
**Results**			
Participants	13*	(a) Report numbers of individuals at each stage of study—eg numbers potentially eligible, examined for eligibility, confirmed eligible, included in the study, completing follow-up, and analysed	Results, sections 0.5-0.7
		(b) Give reasons for non-participation at each stage	N/A
		(c) Consider use of a flow diagram	N/A
Descriptive data	14*	(a) Give characteristics of study participants (eg demographic, clinical, social) and information on exposures and potential confounders	Results, sections 0.5
		(b) Indicate number of participants with missing data for each variable of interest	N/A
Outcome data	15*	Report numbers in each exposure category, or summary measures of exposure	Results, sections 0.5

Main results	16	(*a*) Give unadjusted estimates and, if applicable, confounder-adjusted estimates and their precision (eg, 95% confidence interval). Make clear which confounders were adjusted for and why they were included	12-13
		(*b*) Report category boundaries when continuous variables were categorized	7
		(*c*) If relevant, consider translating estimates of relative risk into absolute risk for a meaningful time period	N/A
Other analyses	17	Report other analyses done—eg analyses of subgroups and interactions, and sensitivity analyses	13-14
**Discussion**			
Key results	18	Summarise key results with reference to study objectives	14
Limitations	19	Discuss limitations of the study, taking into account sources of potential bias or imprecision. Discuss both direction and magnitude of any potential bias	16-17
Interpretation	20	Give a cautious overall interpretation of results considering objectives, limitations, multiplicity of analyses, results from similar studies, and other relevant evidence	14-18
Generalisability	21	Discuss the generalisability (external validity) of the study results	17
**Other information**			
Funding	22	Give the source of funding and the role of the funders for the present study and, if applicable, for the original study on which the present article is based	24

*Give information separately for cases and controls.

Note: An Explanation and Elaboration article discusses each checklist item and gives methodological background and published examples of transparent reporting. The STROBE checklist is best used in conjunction with this article (freely available on the Web sites of PLoS Medicine at http://www.plosmedicine.org/, Annals of Internal Medicine at http://www.annals.org/, and Epidemiology at http://www.epidem.com/). Information on the STROBE Initiative is available at http://www.strobe-statement.org.
