# Supplementary material for: Co-exposure to planned power outages and wildfire PM2.5 and acute healthcare utilization in California: A time-stratified case-crossover study
Source: PLoS Med. 2026 Oct 27;23(10):e1005270. doi: 10.1371/journal.pmed.1005270 (PMC13712598; doi:10.1371/journal.pmed.1005270)
Supplement: Supplementary file 2 [file pmed.1005270.s002.pdf]

S1 Methods.

Circuit-level data description.

The PSPS dataset contains circuit-level information for each PSPS event across California's three major utilities (Pacific Gas and Electric Company [PG&E], San Diego Gas & Electric [SDG&E], and Southern California Edison [SCE]). A circuit is a network of electrical lines that delivers electricity from a power source to a defined service area of customers. A PSPS event may impact multiple circuits, so each circuit-event had a start time, end time, duration, and number of residential and commercial customers impacted. The circuits were provided as geospatial polygons with an 18-meter buffer around the circuit polyline that was approximated to include all customers potentially served by that circuit. The PSPS dataset contained the number of customers affected by each outage along the circuit. The data contained information on both commercial and residential customers.

We overlaid these circuits with high-resolution gridded estimates of population from the 2020 census in California to estimate (1) the number of customers theoretically served by the circuit, and (2) the number of customers affected and the number of people served at the zip code level. In the absence of more detailed information on customer distribution, we assumed an even distribution of customers across the circuit and performed two spatial overlays of the circuits with the gridded population data. The first used the number of customers impacted on a circuit and split them across zip codes proportional to the overlapped area between the zip code and the circuit, to calculate the number of customers impacted during a circuit-event for each zip code. The second used the population pixels that intersected with each circuit to determine the population served by the circuit and split them across zip codes in the same way to calculate the number of customers served by a circuit for each zip code. By summing the corresponding numbers across all PSPS circuit-events by zip code, we calculated the number of customers impacted and the population served on each zip code for each PSPS event. Notably, the population served (denominator) remained constant from event to event, while the customers impacted (numerator) changed.

This analysis includes approximately 1,500 zip codes, most of which intersect with several circuits (median = 11, mean = 15; 93% intersect $\geq$2 circuits). Additionally, most circuits span multiple zip codes (median = 2; 31% span $\geq$3 zip codes). In areas included in this study, the median zip code population ($\sim$10,700) is about five times the median circuit population ($\sim$2,000).

Absolute (primary) and relative (secondary) PSPS exposure metrics.

We derived and included two daily-zip code exposure metrics meant to capture different aspects of PSPS events. The first metric was the total number of customers impacted in each zip code (absolute metric). Because of highly variable population counts within zip codes, we also constructed a secondary relative metric meant to measure the severity of the PSPS event. The secondary metric was computed by multiplying the total number of customers without power (absolute metric) by the proportion of customers affected in each zip code (total number of customers without power divided by total customers served) to generate the relative metric. This metric was meant to capture both the total number of customers out (as represented in the absolute metric) and how widespread the power outage was in the zip code – as both aspects of the power outage could influence healthcare utilization. This proportion was calculated using the numerator and denominator described in the *Circuit-level data description*. Exposure for both metrics was categorized as no exposure or mild, moderate, or severe exposure based on rounded tertiles of the non-zero exposure values of each metric for each event-zip code. For

our primary metric, the magnitude of same-day PSPS events were classified as mild when $\leq$ 61 customers were without power, moderate when the number of customers without power was $>$ 61 and $\leq$ 593, and severe when $>$ 593 customers were without power. For our secondary metric, magnitude for same-day events was again classified as mild (when our relative metric was $\leq$ 1), moderate (1 $<$ relative metric $\leq$ 145), or severe (when our relative metric was $>$ 145). For our primary metric, the magnitude of $\text{lag}_{0\text{-}3}$ PSPS events were classified as mild when $\leq$ 45 customers were without power, moderate when the number of customers without power was $>$ 45 and $\leq$ 458, and severe when $>$ 458 customers were without power. For our secondary metric, magnitude for $\text{lag}_{0\text{-}3}$ events was again classified as mild (when our relative metric was $\leq$ 1), moderate (1 $<$ relative metric $\leq$ 139), or severe (when our relative metric was $>$ 139). These tertiles were calculated at the event-zip code level to ensure equal weighting of longer and shorter events and retain the basis of the severity evaluation being on number of people rather than duration.

Given the variability in zip code size, these absolute and relative metrics enabled us to measure exposure to PSPS outages, both in terms of the actual number of customers affected (absolute) and in terms of the portion of customers affected (relative). We leveraged the absolute metric for the primary analysis, and used our relative metric for a secondary.

The PSPS dataset included customers rather than individual people impacted. Customers impacted included both commercial and residential customers, meaning businesses and residences (e.g., individual apartments, single-family residences). This means that our primary exposure metric reflected the number of customers affected, not the number of people affected. For our secondary relative metric, the numerator included customers impacted, while the denominator relied on gridded population data representing individual people. This meant we consistently underestimated the proportion of people without power, but we do not expect this to be differential with respect to outcomes.

Model equation.

Regression Equation

$$\log\left(\frac{p_{ij}}{1-p_{ij}}\right) = \alpha_i + \beta_1 \cdot PSPS_{ij} + \beta_2 \cdot PM_{2.5,ij} + \beta_3 \cdot (PSPS_{ij} \times PM_{2.5,ij}) + f(Temp_{max,ij}) \quad (1)$$

where: $p_{ij} = P(Y_{ij} = 1|X_{ij}, C_{ij})$ for a case $(j = 1)$ or a control $(j = 0)$ in stratum ij. i represents each stratum and j represents a case or control in that stratum.

Parameter Definitions

- α_i: stratum-specific intercept that accounts for the baseline risk of the health outcome in each stratum, or matched set in this case.
- β_1: log odds ratio for emergency department visit or hospital admission associated with exposure to a PSPS event (binary indicator), assuming no wildfire $PM_{2.5}$ exposure and adjusted for temperature
- β_2: log odds ratio for emergency department visit or hospital admission per $10\,\mu g/m^3$ higher wildfire $PM_{2.5}$ concentration, assuming no PSPS event exposure and adjusted for temperature

- β_3: log odds ratio for the multiplicative interaction between PSPS event exposure and wildfire $PM_{2.5}$(per $10\,\mu g/m^3$). This is the additional effect of $10\,\mu g/m^3$ higher wildfire $PM_{2.5}$in the presence of a PSPS event.
- $f(\mathrm{Temp}_{\mathrm{max}})$: natural cubic spline with 3 degrees of freedom for daily maximum temperature.

PSPS event exposure and wildfire $PM_{2.5}$exposure represent either same-day or $lag_{0\text{-}3}$, for each of the two main analyses – the timing of the two exposures is always parameterized consistently. The joint effect of a PSPS event and $10\,\mu g/m^3$ higher $PM_{2.5}$exposure was calculated as: $\beta_1 + \beta_2 + \beta_3$.

Secondary analysis using outage durations.

While we omitted duration from our primary analysis, longer PSPS events are likely to have greater health impacts. To assess this, we conducted a secondary analysis where we used number of hours without power in the prior week, irrespective of the number of affected households, as our primary PSPS exposure.

To calculate daily PSPS outage duration by zip code, we expanded each PSPS event to hourly intervals based on its start and end times. We rounded the start time down and the end time up to the nearest hour. We then de-duplicated overlapping hours across events within the same zip code to avoid double-counting hours when multiple PSPS events affected the same zip code during the same hour. Finally, we aggregated the unique outage hours to the daily level in order to generate the number of hours out per zip code-day (maximum 24 hours per day due to no double-counting hours). We then summed the number of hours without power in the week prior to index or referent day and used this as the PSPS exposure in our models.
