# Supplementary material for: Co-exposure to planned power outages and wildfire PM2.5 and acute healthcare utilization in California: A time-stratified case-crossover study
Source: PLoS Med. 2026 Oct 27;23(10):e1005270. doi: 10.1371/journal.pmed.1005270 (PMC13712598; doi:10.1371/journal.pmed.1005270)
Supplement: Supplementary file 3 [file pmed.1005270.s003.pdf]

S1 Table. ICD-9 and ICD-10 codes used to define health endpoints. *International Classification of Disease, Ninth Edition* (*ICD*-9) and *Tenth Edition* (*ICD*-10) codes used in analysis, by medical condition. COPD, chronic obstructive pulmonary disease.

**Health endpoint**	***ICD-9 Codes***	***ICD-10 Codes***
Respiratory	460-466, 471, 472, 477, 478, 480-487, 490-496, 511, 513-519, 786	J00-J06, J12-J18, J20-J22, J30, J31, J33, J34, J38, J39, J40-J47, J80-J86, J90-J92, J94, J96-J99, R04-R07, R09
COPD	491-492, 496	J41-44
Cardiovascular	390-398, 401-417, 420-429, 440-449	I00-I02, I05-I16, I20-I28, I30-I5A, I70-I79
Psychiatric	290-293, 295, 296, 298	F06, F10-F16, F18-F21, F23, F25, F28-F33
