# Supplementary material for: Co-exposure to planned power outages and wildfire PM2.5 and acute healthcare utilization in California: A time-stratified case-crossover study
Source: PLoS Med. 2026 Oct 27;23(10):e1005270. doi: 10.1371/journal.pmed.1005270 (PMC13712598; doi:10.1371/journal.pmed.1005270)
Supplement: Supplementary file 4 [file pmed.1005270.s004.pdf]

S2 Table. Demographic characteristics of included emergency department visits and hospitalizations. Demographic characteristics of emergency department visits or hospitalizations included in the study in California from 2013–2019. Each observation represents a single visit, so individuals with multiple visits appear multiple times. COPD, chronic obstructive pulmonary disease.

	All-cause respiratory	COPD	Cardiovascular	Psychiatric
**Age Group**				
20-49 years	2,799,372 (41.9%)	33,415 (6.5%)	434,901 (14.9%)	1,267,283 (65.2%)
50-64 years	1,816,130 (27.2%)	182,937 (35.4%)	827,568 (28.4%)	509,478 (26.2%)
65-79 years	1,307,829 (19.6%)	197,598 (38.3%)	938,150 (32.2%)	139,455 (7.2%)
80 years and older	759,490 (11.4%)	102,477 (19.8%)	712,719 (24.5%)	27,493 (1.4%)
**Sex**				
Female	3,831,871 (57.3%)	277,053 (53.6%)	1,368,501 (47%)	779,186 (40.1%)
Male	2,850,703 (42.7%)	239,351 (46.3%)	1,544,695 (53%)	1,164,301 (59.9%)
Unknown, missing, or invalid	247 (0%)	23 (0%)	142 (0%)	222 (0%)
**Race/Ethnicity**				
Non-Hispanic Black	904,853 (13.5%)	75,193 (14.6%)	334,157 (11.5%)	253,437 (13%)
Native Hawaiian or Other Pacific Islander	464,021 (6.9%)	26,358 (5.1%)	262,388 (9%)	81,212 (4.2%)
Non-Hispanic White	2,907,307 (43.5%)	326,264 (63.2%)	1,489,710 (51.1%)	999,038 (51.4%)
Other or Multiracial	251,544 (3.8%)	13,313 (2.6%)	110,041 (3.8%)	77,130 (4%)
Hispanic	2,057,857 (30.8%)	68,556 (13.3%)	679,615 (23.3%)	491,626 (25.3%)
American Indian or Alaska Native	31,678 (0.5%)	2,945 (0.6%)	11,538 (0.4%)	9,865 (0.5%)
Unknown	65,561 (1%)	3,798 (0.7%)	25,889 (0.9%)	31,401 (1.6%)
