# Supplementary material for: Co-exposure to planned power outages and wildfire PM2.5 and acute healthcare utilization in California: A time-stratified case-crossover study
Source: PLoS Med. 2026 Oct 27;23(10):e1005270. doi: 10.1371/journal.pmed.1005270 (PMC13712598; doi:10.1371/journal.pmed.1005270)
Supplement: Supplementary file 5 [file pmed.1005270.s005.pdf]

S3 Table. Index days by same-day exposure status. Index days are days when a healthcare encounter for all-cause respiratory, COPD, cardiovascular, or psychiatric endpoints occurred. Wildfire $PM_{2.5}$exposure represents the mean wildfire $PM_{2.5}$concentration on that day. PSPS event magnitude (mild/moderate/severe) were classified using tertiles for both absolute and relative metrics. For our absolute (primary) metric, mild events were those where ≤ 61 customers were without power, moderate were those where the number of customers without power was > 61 and ≤ 593, and severe was defined as events where > 593 customers were without power. For our relative (secondary) metric, magnitude was classified as mild when our relative metric was ≤ 1, moderate when $1 <$ relative metric ≤ 145, or severe when our relative metric was > 145. COPD, chronic obstructive pulmonary disease; PSPS, public safety power shutoff.

	Absolute (primary)	Relative (secondary)
**Disease endpoint**	**Index days**	**Index days**
**All-cause respiratory**		
**Exposure (PSPS)**		
Mild	2,134	2,907
Moderate	2,050	2,429
Severe	4,053	2,901
None	6,674,584	6,674,584
**Exposure (wildfire $PM_{2.5}$)**		
Mean (SD), µg/m^3		0.47 (4.09)
**COPD**		
**Exposure (PSPS)**		
Mild	190	248
Moderate	217	256
Severe	558	461
None	515,462	515,462
**Exposure (wildfire $PM_{2.5}$)**		
Mean (SD), µg/m^3		0.53 (4.67)
**Cardiovascular**		
**Exposure (PSPS)**		
Mild	1,396	1,885
Moderate	1,311	1,582
Severe	2,597	1,837
None	2,908,034	2,908,034
**Exposure (wildfire $PM_{2.5}$)**		
Mean (SD), µg/m^3		0.48 (3.94)
**Psychiatric**		
**Exposure (PSPS)**		
Mild	635	841
Moderate	610	727
Severe	1,031	708
None	1,941,433	1,941,433
**Exposure (wildfire $PM_{2.5}$)**		
Mean (SD), µg/m^3		0.49 (3.9)
