# Supplementary material for: Co-exposure to planned power outages and wildfire PM2.5 and acute healthcare utilization in California: A time-stratified case-crossover study
Source: PLoS Med. 2026 Oct 27;23(10):e1005270. doi: 10.1371/journal.pmed.1005270 (PMC13712598; doi:10.1371/journal.pmed.1005270)
Supplement: Supplementary file 6 [file pmed.1005270.s006.pdf]

S4 Table. Index days by lag$_{0\text{-}3}$ day exposure status. Index days are days when a healthcare encounter for all-cause respiratory, COPD, cardiovascular, or psychiatric endpoints occurred. Wildfire $PM_{2.5}$exposure represents the mean wildfire $PM_{2.5}$concentration for lag$_{0\text{-}3}$ days. PSPS event magnitude (mild/moderate/severe) were classified using tertiles for both absolute and relative metrics. For our absolute (primary) metric, mild events were those where $\leq$ 45 customers were without power, moderate were those where the number of customers without power was $>$ 45 and $\leq$ 458, and severe was defined as events where $>$ 458 customers were without power. For our relative (secondary) metric, magnitude was classified as mild when our relative metric was $\leq$ 1, moderate when 1 $<$ relative metric $\leq$ 139, or severe when our relative metric was $>$ 139. COPD, chronic obstructive pulmonary disease; PSPS, public safety power shutoff.

	Absolute (primary)	Relative (secondary)
**Disease endpoint**	**Index days**	**Index days**
**All-cause respiratory**		
**Exposure (PSPS)**		
Mild	2,434	3,126
Moderate	2,547	3,077
Severe	4,666	3,444
None	6,673,174	6,673,174
**Exposure (wildfire $PM_{2.5}$)**		
Mean (SD), µg/m^3		0.47 (3.7)
**COPD**		
**Exposure (PSPS)**		
Mild	223	273
Moderate	271	337
Severe	617	501
None	515,316	515,316
**Exposure (wildfire $PM_{2.5}$)**		
Mean (SD), µg/m^3		0.52 (4.13)
**Cardiovascular**		
**Exposure (PSPS)**		
Mild	1,604	2,037
Moderate	1,637	2,059
Severe	3,095	2,240
None	2,907,002	2,907,002
**Exposure (wildfire $PM_{2.5}$)**		
Mean (SD), µg/m^3		0.48 (3.54)
**Psychiatric**		
**Exposure (PSPS)**		
Mild	720	933
Moderate	745	900
Severe	1,212	844
None	1,941,032	1,941,032
**Exposure (wildfire $PM_{2.5}$)**		
Mean (SD), µg/m^3		0.49 (3.51)
