# Supplementary material for: Co-exposure to planned power outages and wildfire PM2.5 and acute healthcare utilization in California: A time-stratified case-crossover study
Source: PLoS Med. 2026 Oct 27;23(10):e1005270. doi: 10.1371/journal.pmed.1005270 (PMC13712598; doi:10.1371/journal.pmed.1005270)
Supplement: Supplementary file 8 [file pmed.1005270.s008.pdf]

S6 Table. Unadjusted association between PSPS events and healthcare utilization. Odds ratios and 95% confidence intervals for the unadjusted association between zip code-level severe PSPS events and all-cause respiratory-, COPD-, cardiovascular-, and psychiatric-related emergency department visits and hospital admissions in California from 2013–2019. Estimates are from four separate conditional logistic regression models. All models include PSPS event exposure, $lag_{0\text{-}3}$ day wildfire $PM_{2.5}$ exposure, and an interaction term between PSPS event and wildfire $PM_{2.5}$ exposure. COPD, chronic obstructive pulmonary disease; PSPS, public safety power shutoff.
**Note: The estimate for the interaction effect represents the additional multiplicative effect of joint exposure to wildfire $PM_{2.5}$ and PSPS event on the outcome of interest. It should not be interpreted as an odds ratio in isolation, but is rather a ratio of odds ratios.*

	Effect estimates, OR (95% CI)			
	**PSPS**	**WF $PM_{2.5}$ (per 10 µg/m³)**	**Joint effect**	**Multiplicative interaction***
**All-cause respiratory**				
Same day	1.06 (1.02, 1.11)	1.01 (1.01, 1.01)	1.16 (1.10, 1.22)	1.08 (1.02, 1.14)
Lag_{0-3}	1.00 (0.96, 1.05)	1.02 (1.01, 1.02)	1.14 (1.07, 1.21)	1.12 (1.04, 1.20)
**COPD**				
Same day	1.29 (1.15, 1.44)	1.02 (1.01, 1.03)	1.37 (1.19, 1.57)	1.05 (0.91, 1.20)
Lag_{0-3}	1.14 (1.02, 1.27)	1.02 (1.01, 1.02)	1.36 (1.15, 1.60)	1.18 (0.98, 1.41)
**Cardiovascular**				
Same day	0.98 (0.93, 1.03)	1.00 (1.00, 1.01)	0.96 (0.90, 1.03)	0.98 (0.92, 1.05)
Lag_{0-3}	1.00 (0.96, 1.05)	1.00 (1.00, 1.00)	0.96 (0.89, 1.04)	0.96 (0.88, 1.04)
**Psychiatric**				
Same day	0.94 (0.87, 1.02)	1.00 (1.00, 1.00)	0.97 (0.88, 1.07)	1.03 (0.93, 1.14)
Lag_{0-3}	0.98 (0.91, 1.06)	1.00 (0.99, 1.00)	0.97 (0.86, 1.10)	1.00 (0.87, 1.14)
