# Supplementary material for: Co-exposure to planned power outages and wildfire PM2.5 and acute healthcare utilization in California: A time-stratified case-crossover study
Source: PLoS Med. 2026 Oct 27;23(10):e1005270. doi: 10.1371/journal.pmed.1005270 (PMC13712598; doi:10.1371/journal.pmed.1005270)
Supplement: Supplementary file 9 [file pmed.1005270.s009.pdf]

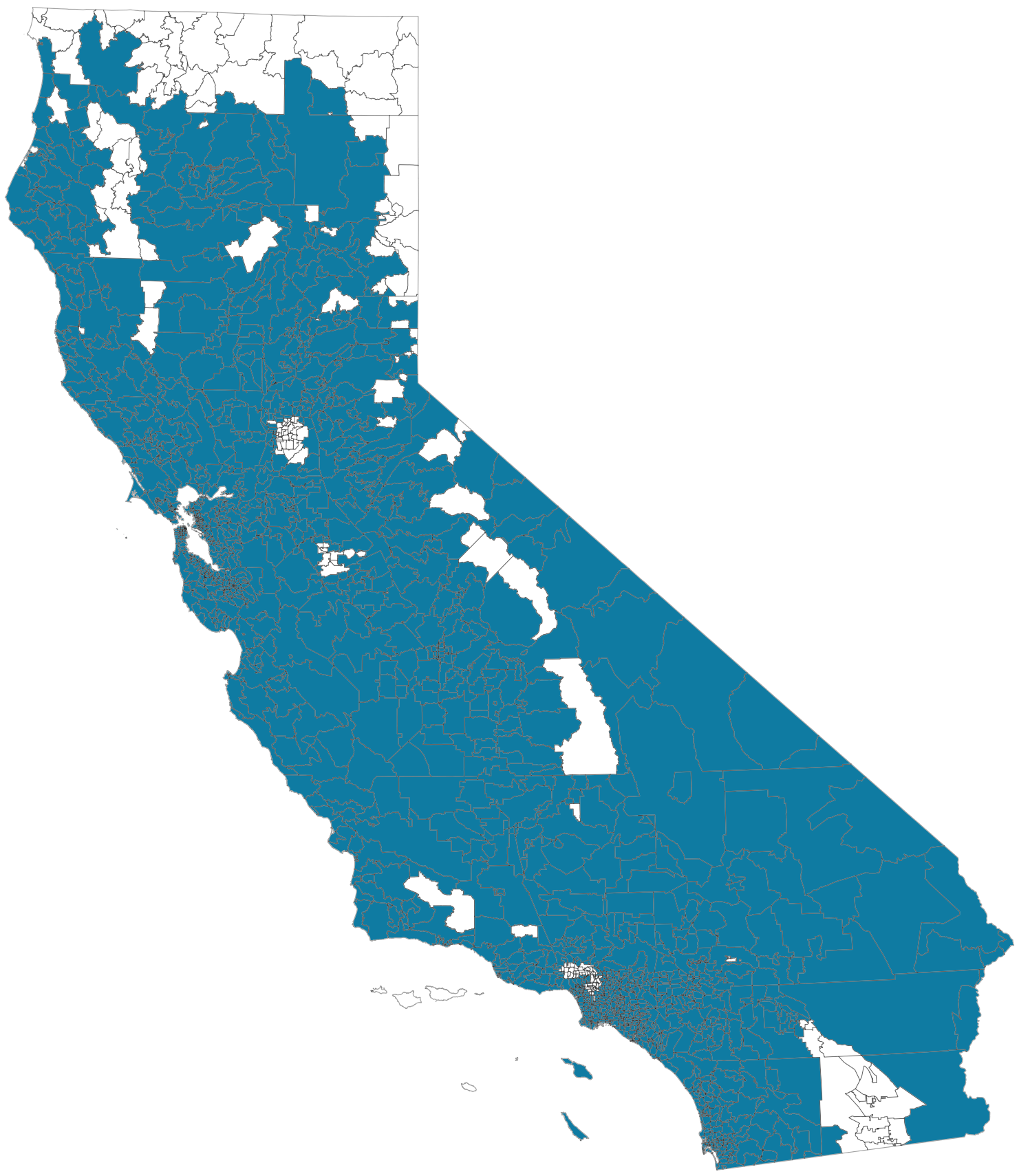

S1 Fig. Zip codes included in the study. Spatial distribution of the 1,504 zip codes in California included in this study. There were 1,504, 1,464, 1,500, 1,501 zip codes included for all-cause respiratory-, COPD-, cardiovascular-, and psychiatric-related emergency department visits and hospital admissions. COPD, chronic obstructive pulmonary disease.
