# Supplementary material for: Co-exposure to planned power outages and wildfire PM2.5 and acute healthcare utilization in California: A time-stratified case-crossover study
Source: PLoS Med. 2026 Oct 27;23(10):e1005270. doi: 10.1371/journal.pmed.1005270 (PMC13712598; doi:10.1371/journal.pmed.1005270)
Supplement: Supplementary file 10 [file pmed.1005270.s010.pdf]

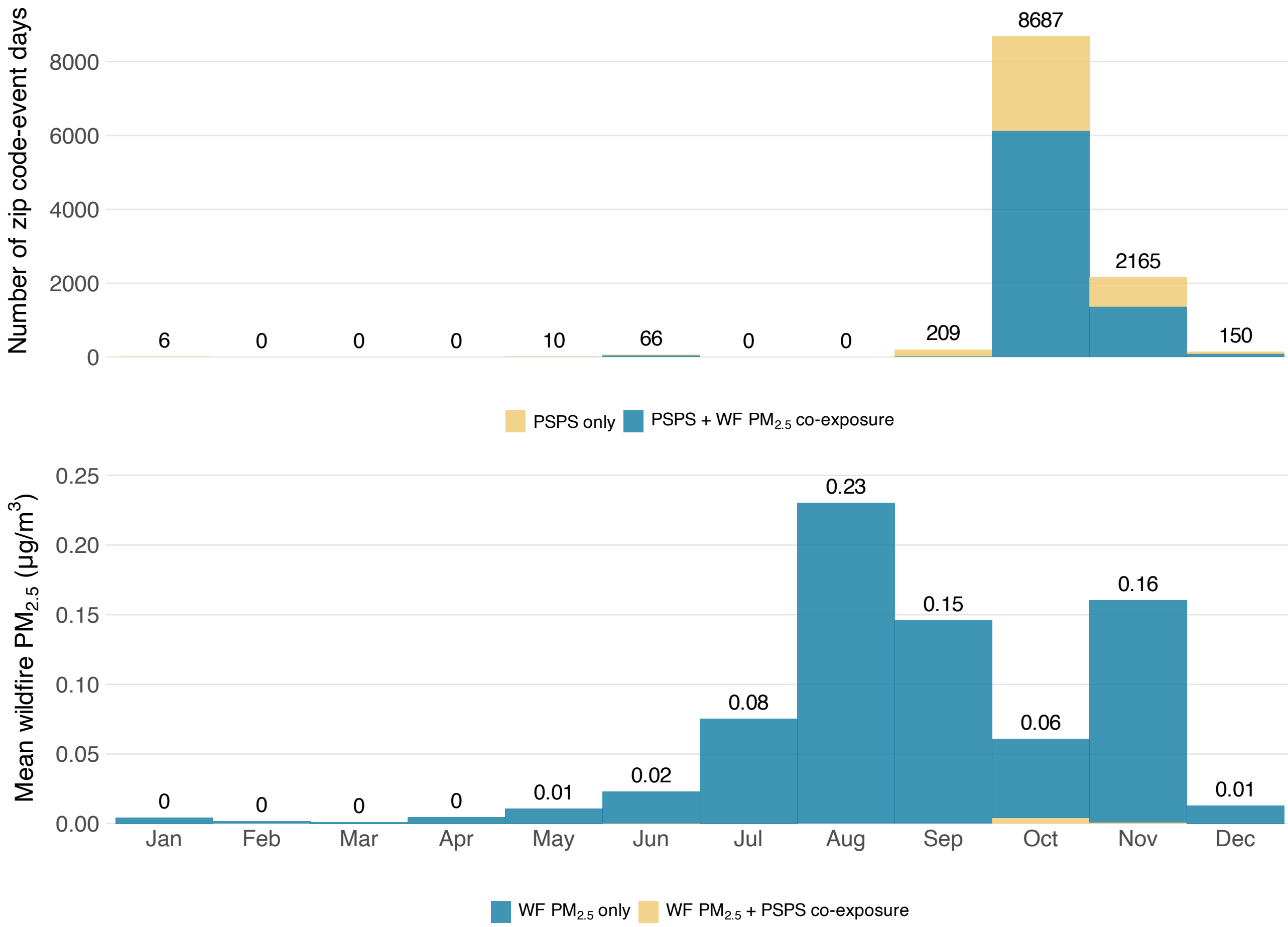


**S2 Fig. Seasonality of PSPS events and wildfire $PM_{2.5}$ exposure.** Number of zip code-event days of PSPS events and mean wildfire $PM_{2.5}$exposure, by month, in California during the study period (2013–2019). The number of zip code-event days of PSPS events reflects the sum over the 7-year period, while the mean wildfire $PM_{2.5}$exposure represents means for each month over the 7-year study period. PSPS, public safety power shutoff.
