# Supplementary material for: Co-exposure to planned power outages and wildfire PM2.5 and acute healthcare utilization in California: A time-stratified case-crossover study
Source: PLoS Med. 2026 Oct 27;23(10):e1005270. doi: 10.1371/journal.pmed.1005270 (PMC13712598; doi:10.1371/journal.pmed.1005270)
Supplement: Supplementary file 14 [file pmed.1005270.s014.pdf]

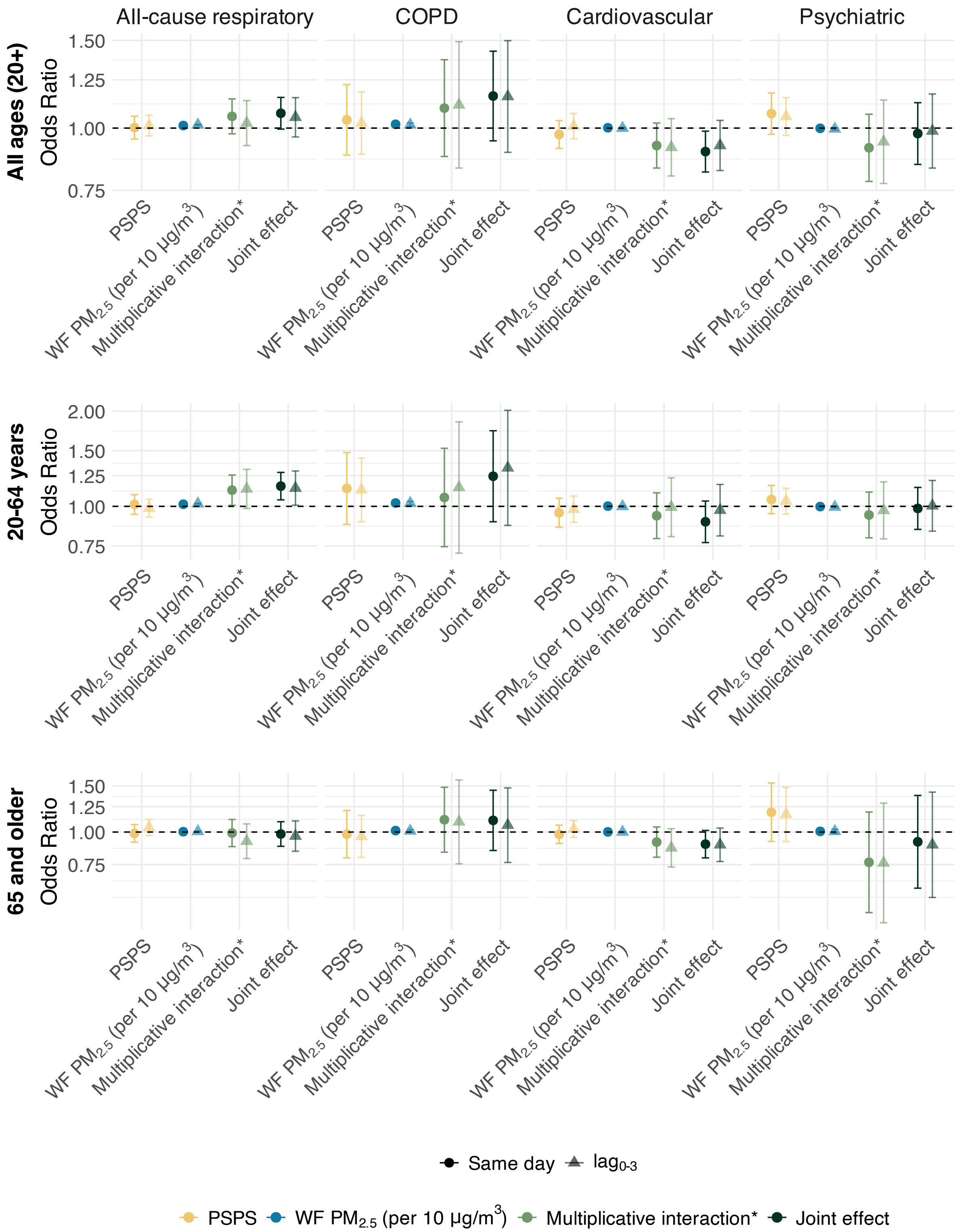


**S6 Fig. Association between moderate PSPS events (relative metric), wildfire PM$_{2.5}$, and healthcare utilization, by age group.** Odds ratios and 95% confidence intervals for the association between zip code-level moderate PSPS events (relative metric) and all-cause respiratory-, COPD-, cardiovascular-, and psychiatric-related emergency department visits and hospital admissions in California from 2013–2019 by age group. Estimates are from eight separate conditional logistic regression models, including one for each health endpoint – exposure metric combination. All models include PSPS event exposure, lag$_{0\text{-}3}$ day wildfire PM$_{2.5}$exposure, an interaction term between PSPS event and wildfire PM$_{2.5}$exposure, and a spline on mean daily temperature. The absolute metric reflects the number of individuals exposed to a PSPS event, while the relative metric reflects the severity of the event.

**Note: The estimate for the interaction effect represents the additional multiplicative effect of joint exposure to wildfire PM$_{2.5}$and PSPS event on the outcome of interest. It should not be interpreted as an odds ratio in isolation, but is rather a ratio of odds ratios.* PSPS, public safety power shutoff; COPD, chronic obstructive pulmonary disease.
